# Emotional Empathy and Facial Mimicry for Static and Dynamic Facial Expressions of Fear and Disgust

**DOI:** 10.3389/fpsyg.2016.01853

**Published:** 2016-11-23

**Authors:** Krystyna Rymarczyk, Łukasz Żurawski, Kamila Jankowiak-Siuda, Iwona Szatkowska

**Affiliations:** ^1^Laboratory of Psychophysiology, Department of Neurophysiology, Nencki Institute of Experimental Biology of Polish Academy of SciencesWarsaw, Poland; ^2^Department of Experimental Psychology, Faculty of Psychology, Institute of Cognitive and Behavioural Neuroscience, University of Social Sciences and HumanitiesWarsaw, Poland

**Keywords:** facial mimicry, empathy, fear, disgust, static, dynamic, facial expressions

## Abstract

Facial mimicry is the tendency to imitate the emotional facial expressions of others. Increasing evidence suggests that the perception of dynamic displays leads to enhanced facial mimicry, especially for happiness and anger. However, little is known about the impact of dynamic stimuli on facial mimicry for fear and disgust. To investigate this issue, facial EMG responses were recorded in the corrugator supercilii, levator labii, and lateral frontalis muscles, while participants viewed static (photos) and dynamic (videos) facial emotional expressions. Moreover, we tested whether emotional empathy modulated facial mimicry for emotional facial expressions. In accordance with our predictions, the highly empathic group responded with larger activity in the corrugator supercilii and levator labii muscles. Moreover, dynamic compared to static facial expressions of fear revealed enhanced mimicry in the high-empathic group in the frontalis and corrugator supercilii muscles. In the low-empathic group the facial reactions were not differentiated between fear and disgust for both dynamic and static facial expressions. We conclude that highly empathic subjects are more sensitive in their facial reactions to the facial expressions of fear and disgust compared to low empathetic counterparts. Our data confirms that personal characteristics, i.e., empathy traits as well as modality of the presented stimuli, modulate the strength of facial mimicry. In addition, measures of EMG activity of the levator labii and frontalis muscles may be a useful index of empathic responses of fear and disgust.

## Introduction

The term facial mimicry (FM) describes the automatic (Dimberg and Thunberg, 1998) and unintentional imitation of emotional expressions in human faces. An increasing number of the studies have argued that FM may be dependent on many particular factors (for review see Hess and Fischer, 2013; Seibt et al., 2015), including the type of task the participant is engaged in Korb et al. (2010) and Murata et al. (2016), properties of the stimulus (dynamic vs. static presentation) (Weyers et al., 2006; Sato et al., 2008; Rymarczyk et al., 2011, 2016) and personal characteristics of the perceiver (e.g., empathic traits) (Sonnby-Borgström et al., 2003; Dimberg et al., 2011; Balconi and Canavesio, 2016). Recently, it has been shown that hormonal level, i.e., administration of oxytocin (Korb et al., 2016) as well as cultural norms (Wood et al., 2016) influence FM expression.

Dynamic facial expressions resemble those that occur in everyday life, so they constitute a powerful medium for emotional communication compared to static expressions, which are mostly used in EMG studies using passive paradigms (Lundquist and Dimberg, 1995; Dimberg et al., 2000). There is considerable evidence that dynamic information is beneficial for various aspects of face processing, e.g., emotion recognition or judgments of intensity and arousal (for review see Krumhuber et al., 2013). Moreover, some studies have reported stronger emotion-specific responses to dynamic as opposed to static expressions, mainly the zygomaticus major muscle (ZM) (Weyers et al., 2006; Rymarczyk et al., 2011) and the corrugator supercilii muscle (CS) for happiness and anger (Sato et al., 2008), respectively; however, the available data is not consistent (for review see Seibt et al., 2015). This may be associated with the different methodologies that were used, e.g., different kinds of stimuli used across studies. Most published studies used some kind of artificial stimuli, e.g., dynamic avatars (Weyers et al., 2006) or image morphing to generate a videos of faces changing from a neutral to emotional expressions (Sato et al., 2008; Rymarczyk et al., 2011). In reference to recent work (Reinl and Bartels, 2015), it could be argued that such stimuli do not contain natural temporal asymmetry typical of authentic emotional facial expressions. These authors reported that “deviations from the natural timeline of expressions lead to a reduction of perceived emotional intensity as well convincingness, and to an increase of perceived artificialness of the dynamic facial expression” (Reinl and Bartels, 2015). In our previous EMG study (Rymarczyk et al., 2016), we used authentic stimuli, i.e., videos showing the emotional facial expressions of actors, and found that subjects responded with stronger EMG activity in the ZM and orbicularis oculi (OO) for dynamic, compared to static displays of happiness, and conclude that the subjects experienced positive emotions. In line with this, neuroimaging data (Trautmann et al., 2009; Arsalidou et al., 2011; Kessler et al., 2011) has revealed that the perception of dynamic compared to static stimuli engaged not only motor- (e.g., inferior frontal gyrus) (Carr et al., 2003), but also brain regions associated with emotion (e.g., amygdala, insula). These regions are also considered to be part of the extended mirror neuron system (MNS) (van der Gaag et al., 2007; Likowski et al., 2012), a neuronal network linked to empathy (Jabbi and Keysers, 2008; Decety, 2010a; Decety et al., 2014).

There is ongoing debate over whether facial mimicry and emotional empathy are associated phenomena (Hatfield et al., 1992; McIntosh, 2006). Some investigators have proposed that facial muscle activity provides proprioceptive information, and that facial expressions can influence internal emotional experiences (Hess and Fischer, 2014). Conversely, it has been suggested that the emotional state of the observer may influence the degree of mimicry such that observed expressions congruent with the perceiver’s emotional state are more quickly and easily mimicked (e.g., Niedenthal et al., 2001). Furthermore, it has been shown that emotional empathy, i.e., process when perception of other’s emotions generates the same emotional state in the perceiver (e.g., de Waal, 2008; Jankowiak-Siuda et al., 2015), is related to the magnitude of facial muscle activity (e.g., Sonnby-Borgstrom, 2002; Sonnby-Borgström et al., 2003; Dimberg et al., 2011; Balconi and Canavesio, 2013; Balconi et al., 2014). For example, using static prototypical facial expressions of happiness and anger, Dimberg et al. (2011) reported that high-empathic subjects responded with greater CS activity to angry compared to happy faces and with larger ZM activity to happy faces compared to angry faces. No differences between expressions of emotions in facial muscle activity were found in the low empathic group. The authors concluded that highly empathic people are particularly responsive to facial expressions. Recently, Balconi and Canavesio (2016) confirmed that empathic traits assessed through questionnaires modulate FM. These authors showed that highly empathic subjects were facially more responsive to happiness compared to subjects with low empathic traits, demonstrated by increased activity in ZM and decreased activity in CS. Moreover, they found that highly empathic participants showed general increased CS responses to negative emotions, i.e., anger and fear, compared with happy and neutral faces. Based on these findings, it is reasonable to assume that the ability to react to the emotional expressions in other people constitutes an important aspect of emotional empathy.

Furthermore, many EMG studies support the phenomenon of facial mimicry, however, most have tested mimicry using presentations of happy and angry faces. There is some evidence for specific facial muscle response patterns for other emotions, i.e., fear and disgust, although the evidence is relatively weak (Hess and Fischer, 2014). A number of studies have characterized ‘fear mimicry’ by increased activity of the CS (Lundquist and Dimberg, 1995; Magnee et al., 2007; Magnée et al., 2007; Balconi et al., 2011; van der Schalk et al., 2011; Balconi and Canavesio, 2013). However, the CS response does not appear to be specific for fear since frowning was also associated with angry (Dimberg and Petterson, 2000; Sato et al., 2008), sad (Lundquist and Dimberg, 1995; Likowski et al., 2008; Weyers et al., 2009) and disgusted faces (Lundquist and Dimberg, 1995; Hess and Blairy, 2001). Recently, (Murata et al., 2016) the activity of CS muscle has been associated with six discrete emotions (anger, disgust, fear, happiness, sadness, and surprise) when participants watched facial expressions as well as when they were specifically instructed to infer a target’s emotional state. To the contrast to the CS, activity of LF muscle, which draws the eyebrows up, was indexed as being typical for fear mimicry. Moreover, little is known about FM for disgust. It appears that apart from CS (Balconi and Canavesio, 2013) and OO (Wolf et al., 2005), levator labii (LL) which creates wrinkles on both sides of the nose, is indexed for “disgust mimicry” (Vrana, 1993; Lundquist and Dimberg, 1995; Cacioppo et al., 2007). The activity of LL during the mimicry of disgusted facial expressions has been reported only in a few studies (for review see Seibt et al., 2015).

The present study has two main aims. Firstly, we assessed whether there is an emotion-specific facial mimicry for fear and disgust facial expressions. Secondly, to examine whether modality of the stimuli (static vs. dynamic) and emotional empathy modulates the strength of FM in a specific setting. Facial EMG responses were measured from three muscles, the CS, LL, and lateral frontalis (LF), while the participants passively viewed static and dynamic displays. We played videos presenting emotional facial expressions of actors. Actors were chosen because of their proficiency in expressing emotional signals (Ekman and Rosenberg, 2005). Based on earlier EMG findings showing that the CS activates during the perception of various negative emotions (e.g., Murata et al., 2016), we did not expect between-emotion-specific activity of this muscle for fear and disgust displays. However, we assumed that emotion-specific activity occur, i.e., the LF activity for fear and LL for disgust. Regarding to modality of the stimuli, we hypothesized that the perception of dynamic compared to static displays, would lead to enhanced FM in all the examined muscles, in particular to the increased activity of the LF for fear and the LL for disgust. In the light of published studies regarding a link between empathy and facial mimicry, we expected that high compared to low-scoring empathic subjects would elicit stronger facial muscle responses, especially for dynamic stimuli. This study is an original attempt to test whether the stimulus modality, together with empathic traits, modulate the facial mimicry for fear and disgust facial expressions.

## Materials and Methods

### Subjects

Thirty two healthy individuals (14 females, mean age = 24.2 ± 3.7 years) participated in this study. The subjects had normal or corrected to normal eyesight and none of them reported neurological diseases. The study was conducted in accordance with guidelines for ethical research and approved by the Ethics Committee at the University of Social Sciences and Humanities. An informed consent form was signed by each participant after the experimental procedures had been clearly explained.

### Stimuli

We used four videos and four static pictures illustrating facial emotional expressions of disgust and fear. The process of creation and emotional rating of stimuli was described in our previous study (Rymarczyk et al., 2016). Stimuli clips of two actresses and actors were used in the experimental procedure. Static pictures depicted the same characters as presented in dynamic ones. Each stimulus clip presented the human face (shown from the front), starting with a view of the neutral, relaxed face of the model (no emotional expressions visible). Dynamic stimulus presentation lasted 2 s and ended with peak expression of a single emotion as the last frame of the stimulus. This occurred at approximately 1 s and remained visible for another second. Conversely, static stimuli consisted of a single frame of the peak expression and lasted 2 s. Stimuli were 880 pixels in height and 720 pixels in width. Emotional characteristics of the stimuli are provided in the **Table 1**.

**Table 1:** Summary statistics of emotional intensity ratings performed for each of emotional labels content of each dynamic facial expression stimuli.

	Mean (standard deviation) of ratings of emotion intensity						
Content	Anger	Happiness	Sadness	Fear	Disgust	Surprise	*N*
**Average ratings of fear expressions**	1,05 (0,23)	1,20 (0,42)	1,05 (0,25)	3,22 (0,76)	1,21 (0,48)	1,93 (0,49)	380
Female actor #1	1,06 (0,27)	1,01 (0,10)	1,04 (0,22)	3,59 (0,60)	1,16 (0,44)	2,08 (0,49)	385
Female actor #2	1,04 (0,19)	1,01 (0,10)	1,04 (0,21)	3,41 (0,61)	1,11 (0,36)	2,03 (0,42)	405
Male actor #1	1,09 (0,30)	1,82 (0,45)	1,07 (0,28)	2,36 (0,51)	1,19 (0,55)	1,88 (0,43)	362
Male actor #2	1,02 (0,14)	1,01 (0,09)	1,07 (0,27)	3,47 (0,62)	1,38 (0,52)	1,70 (0,53)	366
**Average ratings of disgust expressions**	1,09 (0,30)	1,01 (0,11)	1,23 (0,44)	1,14 (0,36)	3,25 (0,88)	1,19 (0,42)	410
Female actor #1	1,13 (0,34)	1,02 (0,14)	1,23 (0,44)	1,37 (0,50)	2,15 (0,54)	1,45 (0,51)	394
Female actor #2	1,08 (0,31)	1,02 (0,14)	1,03 (0,17)	1,05 (0,25)	3,72 (0,58)	1,14 (0,36)	409
Male actor #1	1,11 (0,33)	1,01 (0,10)	1,60 (0,51)	1,10 (0,32)	3,09 (0,54)	1,08 (0,31)	408
Male actor #2	1,03 (0,18)	1,00 (0,00)	1,06 (0,28)	1,04 (0,22)	3,97 (0,47)	1,11 (0,37)	430

*N denotes number of performed ratings for each stimulus.*

### Procedure

The participants were tested individually, sitting in front of a 19-inch LCD screen in a sound-attenuated room. To disguise the real purpose of the study we informed each participant that sweat gland activity would be measured while they watched the actors selected for commercials by an external marketing company. Participants signed a written consent form and EMG electrodes were attached. Later, to enhance the comfort of the subjects, we asked the participants to complete a dummy questionnaire and verbally encouraged them to behave naturally.

Consistent with the methodology of Dimberg (1982), randomized blocks of eight stimuli were presented. Participants were asked to passively view stimuli on a gray background in the center of a screen. In each block pparticipants saw either fear or disgust expressions, each of eight stimuli was either static or dynamic. In other words, four kinds of blocks were created (disgust static, disgust dynamic, fear static, and fear dynamic). Each display started with a white fixation cross, 80 pixels in diameter, appearing for 2 s accompanied by a sound (standard windows reminder – ding.wav). Inter-stimulus intervals with a blank gray screen lasted 8.75–11.25 s. Within each block, randomized stimuli of two opposite-sex pairs of each trial type were presented. No facial expression from the same actor was shown consecutively and within each block each stimulus was repeated once. In summary, each stimulus was shown four times within each block, for a total of 16 presentations within each condition.

After the recording session, the participants completed the questionnaires assessing empathy. The Questionnaire Measure of Emotional Empathy (QMEE) developed by Mehrabian and Epstein (1972) was used. The QMEE contains 33-items to be completed using a 9-point ratings from -4 (=very strong disagreement) to +4 (=very strong agreement). The authors defined empathy as “a vicarious emotional response to the perceived emotional experiences of others” (Mehrabian and Epstein, 1972, 1). We used a Polish translation of the QMEE that had been recommended for this type of scientific research (Rembowski, 1989). Finally participants completed a demographics questionnaire, and were informed of the real purpose of the study.

### Apparatus

Experimental events were controlled using Presentation^®^ software (version 14.6) running on an IBM computer with Microsoft Windows 7 operating system. Procedure was displayed on a 19-inch LCD monitor (NEC multisync LCD 1990 FX; 1280 pixels × 1024 pixels resolution; 32 bit color rate; 75 Hz refresh rate) from a viewing distance of approximately 65 cm.

### EMG Recording and Analysis

Data were recorded using Ag/AgCl electrodes with a diameter of 4 mm filled with electrode paste. The electrodes were positioned in pairs over three muscles – the CS, LL, and LF- on the left side of the face (Fridlund and Cacioppo, 1986). A reference electrode, 10 mm in diameter, was attached to the forehead. Before the electrodes were attached, the skin was cleaned with alcohol and a thin coating of electrode paste was applied. This procedure was repeated until electrode impedance was reduced to 5 kΩ or less. The EMG signals were recorded using a BrainAmp amplifier (Brain Products) and BrainVision Recorder. The hardware low-pass filtered the signal at 560 Hz. Finally, data was digitized using a 24-bit A/D converter with a sampling rate of 2 kHz, and stored on a computer running MS Windows XP for oﬄine analysis.

The BrainVision Analyser 2 (version 2.1.0.327) re-referenced the data to bipolar measures and filtered it at 30 Hz high-pass, 500 Hz low-pass, and 50 Hz notch filters. After rectification and integration over 125 ms, the signal was resampled to 10 Hz. Artifacts in the data were detected in two ways. Firstly, when single muscle activity was above 8 μV at baseline (visibility of fixation cross), the trial was classified as an artifact and excluded from further analysis. All remaining trials were blind-coded and visually checked for artifacts. Later, trials were baseline corrected such that the EMG response was measured as the difference of averaged signal activity between the stimuli duration (2 s) and baseline period (2 s). Finally, the signal was averaged for each condition for each participant and imported to SPSS 21 for statistical analysis.

For testing differences in EMG responses, a two-way repeated-measures ANOVAs with two within-subjects factors (emotion: disgust, fear; stimulus modality: static, dynamic) and one between-subjects factor [emotional empathy: high empathy (HE), low empathy (LE)] were used. Between-subjects factor was created by dividing the subjects according to their median score of QMEE questionnaire. Separate ANOVAs were calculated for responses from a single muscle. Results were reported with a Bonferroni correction for multiple comparisons. In order to confirm that EMG activity changed from baseline and facial mimicry occurred, the EMG data were tested for a difference from a zero (baseline) using one-sample, two-tailed *t*-tests.

## Results

### Empathy Scores

Subjects were differentiated by their empirically established median score on the QMEE questionnaire into HE and LE groups. The QMEE scores of the two groups were significantly different [*t*_(30)_ = 7.660, *p* = 0.000, *M*_HE_ = 63,440, SE_HE_ = 4,163; *M*_LE_ = 20,500 SE_LE_ = 3,754].

### Corrugator Supercilii

**FIGURE 1:**
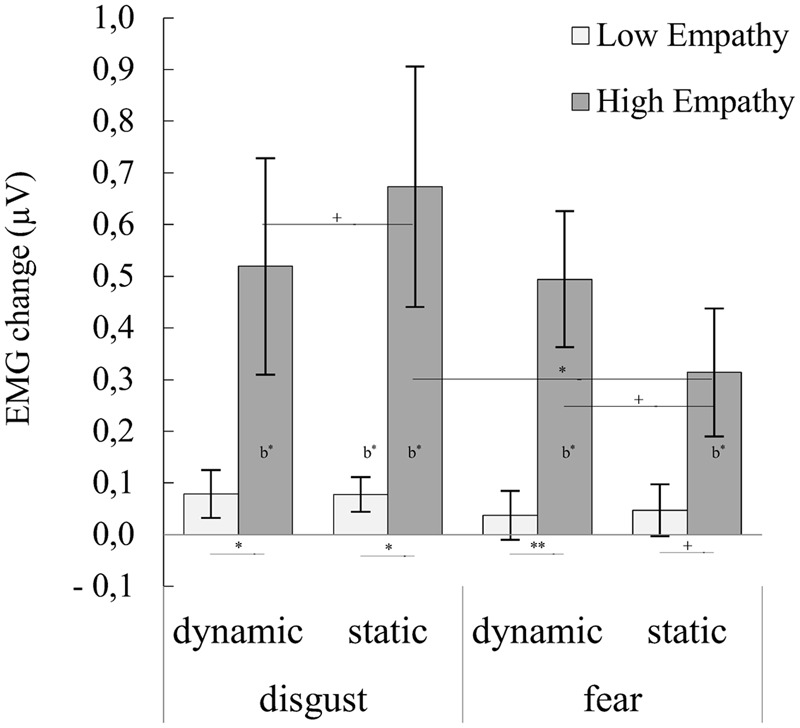
**Mean (±SE) EMG activity changes and corresponding statistics for corrugator supercilii during presentation conditions moderated by empathy groups.** Asterisks with lines beneath indicate significant differences between conditions (simple effects) in EMG responses: ^+^*p* < 0.1, ^∗^*p* < 0.05, ^∗∗^*p* < 0.01. Asterisks followed “b” indicate significant differences from baseline EMG responses: b^∗^*p* < 0.05.

For the CS muscle, ANOVA showed significant main effect of emotional empathy groups [*F*_(**1,30**)_ = 9.440, *p* = 0.004, η^2^ = 0.239]. HE (*M* = 0.500, *SE* = 0.101) compared to LE (*M* = 0.060, *SE* = 0.101) subjects reacted with stronger EMG activity. Significant interactions of emotion × modality [*F*_(**1,30**)_ = 4.353, *p* = 0.046, η^2^ = 0.127] and emotion x modality × emotional empathy groups [*F*_(**1,30**)_ = 4.978, *p* = 0.033, η^2^ = 0.142] were found. The latter interaction showed that (see **Figure 1**, for statistics see **Table 2**; Supplementary Table S1): (1) HE compared with LE people reacted with stronger CS response for dynamic and static disgust as well as for dynamic and static fear facial expressions; (2) HE subjects reacted with stronger CS for static disgust compared to static fear; (3) HE subjects reacted with higher EMG activity for dynamic than static fear expressions (trend effect); (4) HE subjects reacted with higher EMG activity for static than dynamic disgust expressions (trend effect).

**Table 2:** Summary statistics and statistics of EMG activity changes for corrugator supercilii during presentation conditions moderated by empathy groups.

			Corrugator supercilii			
			*M*	*SE*	*t*	*p*
Low empathy group	Disgust	Dynamic	0,079	0,046	1,709	0,108
		Static	0,078	0,034	2,322	0,035
	Fear	Dynamic	0,037	0,047	0,775	0,450
		Static	0,047	0,050	0,936	0,364
High empathy group	Disgust	Dynamic	0,519	0,209	2,479	0,026
		Static	0,673	0,233	2,893	0,011
	Fear	Dynamic	0,494	0,132	3,753	0,002
		Static	0,314	0,124	2,535	0,023

*M, mean; SE, standard error of mean; *t*-value of one sample *t*-test (two-sided) comparing mean value for each condition in table with 0, indicating whether EMG changed from baseline; *p* – significance value of one-sample *t*-test (if *p* < 0,05 values in column “*M*” differ from baseline).*

No significant differences were found for main effect of emotion [*F*_(**1,30**)_ = 1,348, *p* = 0.255, η^2^ = 0.043], modality [*F*_(**1,30**)_ = 0.006, *p* = 0.937, η^2^ = 0.000] as well as no interactions: emotion × emotional empathy groups [*F*_(**1,30**)_ = 0.619, *p* = 0.438, η^2^ = 0.020], modality × emotional empathy groups [*F*_(**1,30**)_ = 0.028, *p* = 0.869, η^2^ = 0.001].

### Levator Labii

**FIGURE 2:**
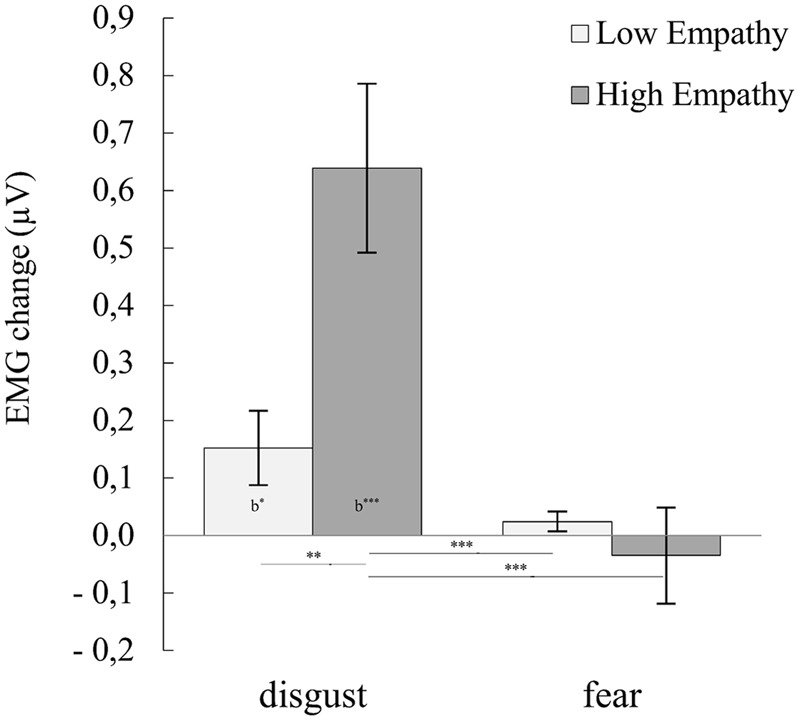
**Mean (±SE) EMG activity changes and corresponding statistics for levator labii in pooled disgust and fear conditions moderated by empathy groups.** Asterisks with lines beneath indicate significant differences between conditions (simple effects) in EMG responses: ^∗∗^*p* < 0.01, ^∗∗∗^*p* < 0.001. Asterisks followed “b” indicate significant differences from baseline EMG responses: b^∗^*p* < 0.05, b^∗∗∗^*p* < 0.001.

For the LL muscle, ANOVA showed significant main effect of emotional empathy groups [*F*_(**1,30**)_ = 6.255, *p* = 0.018, η^2^ = 0.173], emotion [*F*_(**1,30**)_ = 17.405, *p* = 0.000, η^2^ = 0.367] and significant interactions of emotion × emotional empathy groups [*F*_(**1,30**)_ = 8.061, *p* < 0.008, η^2^ = 0.212]. Main effect of emotional empathy groups have shown that HE (*M* = 0.302, *SE* = 0.060) compared to LE (*M* = 0.088, *SE* = 0.060) subjects reacted with stronger LL activity. Main effect of emotion revealed that subjects responded with higher LL activity to disgust than fear. Emotional empathy groups x emotion interaction (see **Figure 2**, for statistics see **Table 3**; Supplementary Table S2) indicated: (1) LL reaction to disgust facial expressions was higher in HE compared to LE subjects; (2) in HE group LL activity was higher for disgust than fear expressions.

**Table 3:** Summary statistics and statistics of EMG activity changes for levator labii in pooled disgust and fear conditions moderated by empathy groups.

		Levator labii			
		*M*	*SE*	*t*	*p*
Low empathy group	Disgust	0,167	0,074	2,347	0,033
	Fear	0,138	0,059	1,411	0,179
High empathy group	Disgust	0,033	0,026	4,353	0,001
	Fear	0,015	0,017	-0,418	0,682

*M, mean; SE, standard error of mean; *t*-value of one sample *t*-test (two-sided) comparing mean value for each condition in table with 0, indicating whether EMG changed from baseline; *p* – significance value of one-sample *t*-test (if *p* < 0,05 values in column “*M*” differ from baseline).*

No significant differences for main effect of modality [*F*_(**1,30**)_ = 0.397, *p* = 0.533, η^2^ = 0.013] were found as well as no interactions: modality × emotional empathy groups [*F*_(**1,30**)_ = 0.949, *p* = 0.338, η^2^ = 0.031], emotion × modality [*F*_(**1,30**)_ = 0.012, *p* = 0.912, η^2^ = 0.000] and emotion × modality × emotional empathy groups [*F*_(**1,30**)_ = 0.016, *p* = 0.900, η^2^ = 0.001].

### Lateral Frontalis

For the LF muscle, ANOVA showed significant main effect of emotion [*F*_(**1,30**)_ = 10.395, *p* = 0.003, η^2^ = 0.257], and significant interactions of emotion × emotional empathy groups [*F*_(**1,30**)_ = 7.805, *p* = 0.009, η^2^ = 0.206], modality × emotional empathy groups [*F*_(**1,30**)_ = 5.098, *p* = 0.031, η^2^ = 0.145] and emotion × modality × emotional empathy groups [*F*_(**1,30**)_ = 5.211, *p* = 0.030, η^2^ = 0.148].

**FIGURE 3:**
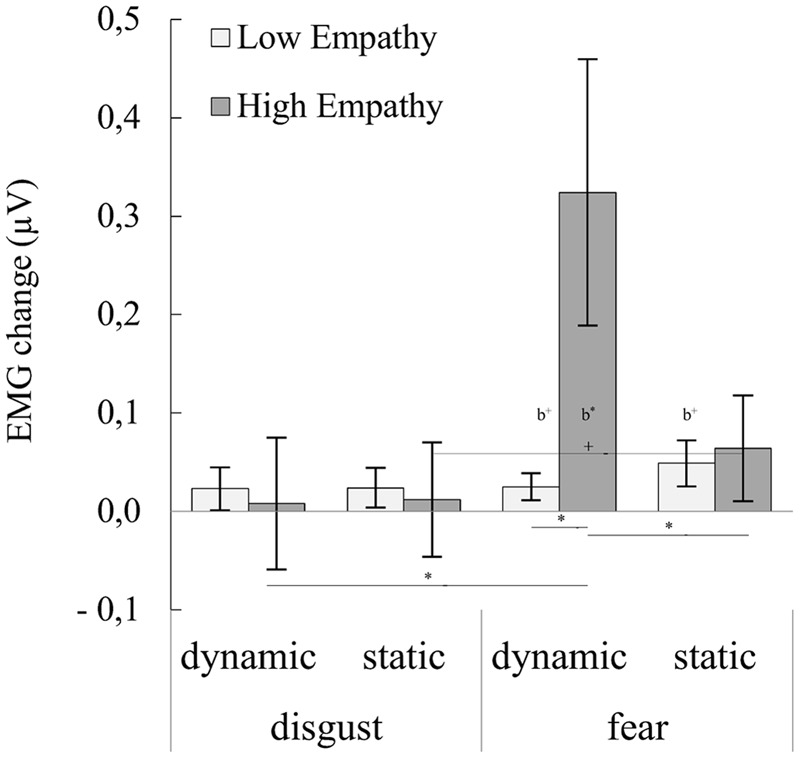
**Mean (±SE) EMG activity changes and corresponding statistics for lateral frontalis during presentation conditions moderated by empathy groups.** Asterisks with lines beneath indicate significant differences between conditions (simple effects) in EMG responses: ^+^*p* < 0.1, ^∗^*p* < 0.05. Asterisks followed “b” indicate significant differences from baseline EMG responses: b^+^*p* < 0.1, b^∗^*p* < 0.05.

Main effect of emotion showed that subjects reacted to fear compared to disgust with stronger LF activity. Emotional empathy groups × emotion interaction showed: (1) higher LF reaction in HE group to fear than disgust facial expressions; (2) HE compared to LE subjects reacted to fear expressions with higher LF activity. Emotional empathy groups × modality interaction indicated that higher LF reaction in HE group to dynamic than static facial expressions. Interaction of emotional empathy groups × emotion × modality showed (see **Figure 3**, for statistics see **Table 4**; Supplementary Table S3): (1) HE compared with LE people reacted with stronger LF response for dynamic fear; (2) HE subjects reacted with stronger LF response for dynamic fear compared to dynamic disgust and with stronger LF activity for static fear compared to static disgust (trend effect); (3) HE subjects reacted with higher EMG activity for dynamic than static fear expressions.

**Table 4:** Summary statistics and statistics of EMG activity changes for lateral frontalis during presentation conditions moderated by empathy groups.

			Lateral frontalis			
			*M*	*SE*	*t*	*p*
Low empathy group	Disgust	Dynamic	0,023	0,022	1,045	0,313
		Static	0,024	0,020	1,224	0,240
	Fear	Dynamic	0,025	0,014	1,817	0,089
		Static	0,049	0,023	2,075	0,056
High empathy group	Disgust	Dynamic	0,008	0,067	0,121	0,905
		Static	0,012	0,058	0,198	0,845
	Fear	Dynamic	0,324	0,135	2,397	0,030
		Static	0,064	0,054	1,178	0,257

*M, mean; SE, standard error of mean; *t*-value of one sample *t*-test (two-sided) comparing mean value for each condition in table with 0, indicating whether EMG changed from baseline; *p* – significance value of one-sample *t*-test (if *p* < 0,05 values in column “*M*” differ from baseline).*

Trend effects were observed for modality factor [*F*_(**1,30**)_ = 3.438, *p* = 0.074, η^2^ = 0.103] as well as well as for interaction of emotion × modality [*F*_(**1,30**)_ = 3.734, *p* = 0.063, η^2^ = 0.111]. Main effect of modality revealed that subjects responded with higher LF activity to dynamic than static facial expressions. Emotion × modality interaction showed: (1) higher LF activity for dynamic than static facial expression of fear; (2) LF activity for dynamic fear was higher than EMG reaction for dynamic disgust; (3) LF activity for static fear was higher than EMG reaction for static disgust.

No significant differences were observed for main effect of emotional empathy groups [*F*_(**1,30**)_ = 1.076, *p* = 0.308, η^2^ = 0.103].

## Discussion

The present study had two aims. First, we assessed whether facial mimicry is found in the emotional expressions of fear and disgust, i.e., we tested emotion-specific activity of the LF muscle for fear and LL for disgust presentations. As we hypothesized we have showed that fear presentations induced activity in the LF muscle, while perception of disgust produced facial activity in the LL muscle, moreover both emotions induced activity of CS muscle. As noted in the introduction, the pattern of increased CS muscle activity for fear and disgust may indicate a contraction of this muscle is associated with negative emotional valence. As well as contraction of the CS muscle associated with anger, fear, disgust or surprise (Murata et al., 2016), contraction of this muscle was observed during disapproval (Cannon et al., 2011) or mental effort (Neumann and Strack, 2000). Thus, the activity of CS could be a general index of global negative affect (Larsen et al., 2003). More importantly, our results demonstrate some emotion–specific patterns of EMG activity, i.e., LF muscle activity for fear and LL muscle activity for disgust. A possible interpretation of fear mimicry is an emotional process indicating fear elicited by a social threat. For example, it has been shown (Moody et al., 2007) that after experiencing fear (watching fear-inducing film clips) subjects presented fearful expressions, as measured by increased frontalis activity. However, to date, it remains to be shown conclusively that activity of the frontalis muscle is a valid indicator of fearful expression. With respect to facial mimicry of disgust, our findings are in line with previous EMG studies demonstrating contraction of the LL muscle during observation of disgust (Vrana, 1993; Lundquist and Dimberg, 1995). Recently, Hühnel et al. (2014) showed increased activity of the LL muscle using dynamic facial expressions of disgust, however, this response was observed only in older compared to younger age group. To conclude, our results suggest that individuals mimic not only smiling and frowning to positive emotions and negative emotions, respectively, but also mimic discrete emotions such as fear and disgust. This supports the theory that facial mimicry is an automatic and innate reflex-like mechanism that is activated in response to emotional states.

Our next goal was to investigate whether stimulus modality and empathic traits are associated with the magnitude of facial muscle activity during mimicry of fear and disgust. As we hypothesized, the HE, compared to LE, group reacted with larger CS activity for all presented conditions. Moreover, in the HE group, change in the activity of the CS muscle was greater in response to dynamic compared to static fear stimuli. The same activity pattern, i.e., a stronger response to dynamic stimuli, was observed in the LF muscle for fear expressions in HE group. On the other hand, the LE group responses were not differentiable between static and dynamic emotions of fear and disgust stimuli in the CS and LF muscles. In the HE group the change in activity of LL was greater in response to disgust compared to fear stimuli regardless of modality (dynamic vs. static) stimuli. In the LE group, the activity of LL was not different between fear and disgust stimuli.

The results concerning empathy traits are in agreement with previous EMG studies, in which highly empathic subjects showed greater mimicry of emotional expressions for happiness and anger (Sonnby-Borgstrom, 2002; Sonnby-Borgström et al., 2003; Dimberg et al., 2011). Recently, it has been shown in highly empathic individuals large amplitude EMG responses were associated with CS muscles not only for facial expression of anger, but also for fear (Balconi and Canavesio, 2016) and disgust (Balconi and Canavesio, 2013). The authors conclude that facial EMG measures may function as a biological marker for the processes associated with to sharing emotion. Our results are broadly in line with the hypothesis (MacDonald, 2003; Dimberg et al., 2011) that automatic mimicry may be a component of emotional empathy.

A recent series of studies examining empathy (Balconi and Canavesio, 2013, 2016) has shown a direct relationship between EMG facial responses and the activity of the prefrontal cortex. Therefore, many neuroimaging studies investigating empathy report that people with higher empathic dispositions have higher activation-levels of empathy-related brain structures such as, the anterior insula (Hein and Singer, 2008), inferior frontal gyrus (Saarela et al., 2006), amygdala (Decety, 2010b) and prefrontal areas (Rameson et al., 2012). Furthermore, it has been shown (Balconi et al., 2011) that temporary inhibition of the medial prefrontal cortex (MPFC) by repeated transcranial magnetic stimulation (rTMS) impairs facial mimicry to angry and fearful faces through the ZM and CS muscles. On the other hand, excitatory high-frequency rTMS of the MPFC enhances mimicry of facial expressions in CS and ZM muscles during an empathic, emotional task (Balconi and Canavesio, 2013). Recently, Korb et al. (2015) have found that inhibition (rTMS) of both right primary motor cortex (M1) and the right primary somatosensory cortex (S1), considered as a part of MNS (for review see Pineda, 2008), also led to reduced facial mimicry. Together, these data suggest that the increased mimicry of facial expressions in highly empathic individuals is mediated by greater activation of empathy-related neural networks.

In our study, EMG responses for facial expressions of fear and disgust were not different in the LE group. A similar finding was reported by Dimberg et al. (2011) for expressions of happiness and anger. It is still an open question whether the lack of EMG activity reflects an inability in this group to both mimic and to react emotionally to facial stimuli. Some explanation comes from a recent study in which BOLD and facial EMG were simultaneously measured in an MRI scanner (Likowski et al., 2012). It was shown that congruent facial reactions recorded from CS and ZM during passive perception of static happy, sad, and angry facial expressions corresponded to activity in prominent parts of the MNS (i.e., the inferior frontal gyrus), as well as areas responsible for emotional processing (i.e., the insula). Thus, the authors suggested that facial mimicry not only involves motor, but affective neural systems simultaneously. Recently, Wood et al. (2016) have proposed that automatic mimicry reflects underlying “sensorimotor simulation” that may support understanding the emotion of others. The authors suggested that processing facial expressions in others activates motor as well as somatosensory neuronal processes involved in producing the facial expression. Moreover, this sensorimotor simulation may elicit an associated emotional state, resulting in accurate understanding of emotion in others. Furthermore, it seems that automatic mimicry does not always occur, e.g., when subject is not motivated to engage in understanding the perceiver (Carr et al., 2014). Therefore, it could be suggested that weaker facial mimicry in low empathy subjects neither imitate facial expressions nor share the emotions of others. On the other hand, it is thought that highly empathic individuals are more likely to imitate and show facial mimicry, because they ‘feel’ the emotions of others. In line with neuroimaging studies examining the perception of facial emotional expressions (van der Gaag et al., 2007; Kessler et al., 2011), it may be assumed that the stronger facial muscle activity in response to dynamic vs. static stimuli may mean that sensorimotor and emotion-related brain structures were activated more strongly in highly empathic subjects. Future studies, such as ones simultaneous measures of BOLD and facial EMG using an MRI scanner with high and low empathic subjects, are warranted to address this issue.

In this study, dynamic stimuli lead to enhanced FM in the HE group only, in particular for expressions of fear in the CS and LF muscles. Contrary to our assumption, the dynamic compared to static disgust displays did not lead to enhanced facial muscle responses in any of the muscles. Moreover, we found that HE subjects elicited stronger CS response for static compared to dynamic disgust representation. This finding is not straightforward to interpret because disgust, similar to fear, conveys information that potentially affects survival (Rozin and Haidt, 2013), so the dynamic modality could be an important factor favoring the avoidance of danger. On the other hand, it has been suggested that fear and disgust often involve divergent mechanisms at the physiological level (Krusemark and Li, 2011). Fear tends to activate sympathetic pathways, prompting the fight-or-flight response, while disgust activates parasympathetic responses, reducing heart rate, blood pressure, and respiration (Ekman et al., 1983). Accordingly, Susskind et al. (2008) reported that subjects have enhanced sensory acquisition (e.g., faster eye movements, air velocity inspiration) when expressing fear, and the opposite pattern associated with facial expressions of disgust. Importantly, both emotions are represented by different neural networks. It has been shown that fear is associated with activation of brain structures involved in the automatic detection of evolutionarily threats, mainly the amygdala (van der Zwaag et al., 2012), while disgust increases activity in the insula, among others structures connected to the sensory domain, i.e., sensation of bad taste (Nieuwenhuys, 2012). Based on the aforementioned studies, it could be debated whether the dynamic modality of stimuli plays a different role in the perception of fear and disgust. It is possible that dynamic fear expressions, in particular, convey higher complexity cues important for avoiding threats. According to this assumption, Hoffmann et al. (2013) have found that fear was more accurately recognized when using dynamic compared to static stimuli, however, the modality factor did not improve recognition in the case of disgust. Some authors have proposed that that certain expressions rely more on motion representation than others (Cunningham and Wallraven, 2009; Fiorentini and Viviani, 2011). To sum up, the stronger mimicry we observed in CS to static compared to dynamic disgust in HE may simply result from an interaction of two factors. Highly empathic people are more prone to mimic facial emotions and/or properties of the stimuli itself, i.e., static images of disgust are more mimicry-engaging than dynamic ones. Further studies are warranted to evaluate the role of stimulus modality and empathy traits in facial mimicry for disgust.

Our findings have partially confirmed the influence of dynamic facial expressions in facial mimicry, i.e., there was increased mimicry for dynamic compared to static fear expressions, especially for the HE group. These results are broadly consistent with the notion that the benefits of using dynamic stimuli arise from the motion representation itself, i.e., unfolding the emotion can provide a stronger intention and enrichment of emotional expressions when compared to static displays (Ambadar et al., 2005). This explanation is in line with neuroimaging findings that have shown that the perception of dynamic facial displays engages brain regions sensitive to processing of emotional stimuli (Kilts et al., 2003), signaling intentions (Gallagher et al., 2000), as well as the MNS, e.g., inferior frontal gyrus (e.g., LaBar et al., 2003; Sato et al., 2004; van der Gaag et al., 2007; Trautmann et al., 2009; Kessler et al., 2011). Based on these finding, we propose that highly empathic subjects, because of their personal characteristics, are particularly sensitive and responsive to the dynamic facial emotional expressions of others.

In summary, our results highlight the importance of the stimulus modality and empathy traits of subjects, in the mimicry of facial expressions for biologically relevant emotions, i.e., fear and disgust facial expressions. Together with others findings, our data confirms an emotion-specific pattern response of the LF for fearful (Lundquist and Dimberg, 1995) and LL for facial expressions of disgust (Vrana, 1993). Consistent with our prediction, there was no between-emotion-specific effect for the CS, thereby indicating that activity of this muscle is generally related to negatively valenced stimuli (Bradley et al., 2001). Our results further show that the EMG recording of the LL and LF provide useful measures of empathic emotional responses. Future studies in natural settings are warranted to understand the mutual links between emotional empathy and FM.

## Author Contributions

Conceived and designed the experiments: KR and ŁŻ. Performed the experiments: KR and ŁŻ. Analyzed the data: KR and ŁŻ. Contributed materials: KR and ŁŻ. Wrote the paper: KR, ŁŻ, KJ-S, and IS.

## Conflict of Interest Statement

The authors declare that the research was conducted in the absence of any commercial or financial relationships that could be construed as a potential conflict of interest.
