# Supplementary material for: Emotional Empathy and Facial Mimicry for Static and Dynamic Facial Expressions of Fear and Disgust
Source: Front Psychol. 2016 Nov 23;7:1853. doi: 10.3389/fpsyg.2016.01853 (PMC5120108; doi:10.3389/fpsyg.2016.01853)
Supplement: Supplementary file 1 [file Table_1.DOCX]

# Supplementary Table 1. Table illustrating mean EMG activity differences for corrugator supercilii during presentation conditions moderated by groups distinguished by emotional empathy score (in interaction of empathy group x emotion x modality).

| **emotion** | **modality** | **empathy group** | ***t*** | ***p*** | **Cohen's *d*** | **meaning of comparison** |
| --- | --- | --- | --- | --- | --- | --- |
| disgust | dynamic | High vs Low | -2,056 | 0,049 | 0,725 | High > Low |
|  | static |  | 2,532 | 0,017 | 0,895 | High > Low |
| fear | dynamic |  | 3,271 | 0,003 | 1,157 | High > Low |
|  | static |  | 2,008 | 0,055 | 0,707 | High > Low |
| disgust vs fear | dynamic | Low | 0,341 | 0,737 | 0,228 | no differences |
|  | static |  | 0,181 | 0,856 | 0,184 | no differences |
|  | dynamic | High | 0,198 | 0,844 | 0,036 | no differences |
|  | static |  | 2,099 | 0,044 | 0,482 | disgust > fear |
| disgust | dynamic vs static | Low | 0,011 | 0,992 | 0,006 | no differences |
| fear |  |  | 0,103 | 0,917 | 0,053 | no differences |
| disgust |  | High | 1,711 | 0,099 | 0,174 | static > dynamic (trend) |
| fear |  |  | 1,856 | 0,074 | 0,353 | dynamic > static (trend) |
