# Supplementary material for: Emotional Empathy and Facial Mimicry for Static and Dynamic Facial Expressions of Fear and Disgust
Source: Front Psychol. 2016 Nov 23;7:1853. doi: 10.3389/fpsyg.2016.01853 (PMC5120108; doi:10.3389/fpsyg.2016.01853)
Supplement: Supplementary file 2 [file Table_2.DOCX]

# Supplementary Table 2. Table illustrating mean EMG activity differences for levator labii in pooled disgust and fear conditions moderated by groups distinguished by emotional empathy score (in interaction of empathy group x modality).

| **emotion** | **empathy group** | ***t*** | ***p*** | **Cohen's *d*** | **meaning of comparison** |
| --- | --- | --- | --- | --- | --- |
| disgust | High vs Low | 3,025 | 0,005 | 1,0719 | High > Low |
| fear |  | 0,686 | 0,494 | 0,2451 | no differences |
| disgust vs fear | Low | 0,941 | 0,354 | 0,6745 | no differences |
|  | High | 4,956 | 0,000 | 1,4099 | disgust > fear |
