# Supplementary material for: Emotional Empathy and Facial Mimicry for Static and Dynamic Facial Expressions of Fear and Disgust
Source: Front Psychol. 2016 Nov 23;7:1853. doi: 10.3389/fpsyg.2016.01853 (PMC5120108; doi:10.3389/fpsyg.2016.01853)
Supplement: Supplementary file 3 [file Table_3.DOCX]

# Supplementary Table 3. Table illustrating mean EMG activity differences for lateral frontalis during presentation conditions moderated by groups distinguished by emotional empathy score (in interaction of empathy group x emotion x modality).

| **emotion** | **modality** | **empathy group** | ***t*** | ***p*** | **Cohen's *d*** | **meaning of comparison** |
| --- | --- | --- | --- | --- | --- | --- |
| disgust | dynamic | High vs Low | 0,214 | 0,835 | 0,074 | no differences |
|  | static |  | 0,210 | 0,836 | 0,074 | no differences |
| fear | dynamic |  | 2,199 | 0,036 | 0,778 | High > Low |
|  | static |  | 0,254 | 0,802 | 0,090 | no differences |
| disgust vs fear | dynamic | Low | 0,024 | 0,979 | 0,030 | no differences |
|  | static |  | 0,857 | 0,397 | 0,277 | no differences |
|  | dynamic | High | 3,807 | 0,001 | 0,741 | fear > disgust |
|  | static |  | 1,857 | 0,074 | 0,231 | fear > disgust (trend) |
| disgust | dynamic vs static | Low | 0,083 | 0,945 | 0,020 | no differences |
| fear |  |  | 0,282 | 0,784 | 0,307 | no differences |
| disgust |  | High | 0,125 | 0,885 | 0,014 | no differences |
| fear |  |  | 3,071 | 0,005 | 0,633 | dynamic > static |
